# Myelin Oligodendrocyte Glycoprotein Antibody-Associated Optic Neuritis Following SARS-CoV-2 Vaccination

**DOI:** 10.7759/cureus.78286

**Published:** 2025-01-31

**Authors:** Bruna Cunha, Pedro Gil, Afonso Murta, Diogo Hipólito-Fernandes, Lívio Costa

**Affiliations:** 1 Ophthalmology, Unidade Local De Saúde De São José, Lisbon, PRT

**Keywords:** coronavirus vaccination, moderna mrna1273, mog-igg-associated optic neuritis, sars-cov-2, unilateral optic neuritis

## Abstract

Myelin oligodendrocyte glycoprotein antibody-associated disease (MOGAD) is an autoimmune demyelinating disorder that occurs in approximately 20% of cases in a postinfectious or postvaccination context. To date, few cases of MOGAD have been reported following SARS-CoV-2 immunization. Here, we report the case of a 28-year-old female presenting with isolated unilateral optic neuritis, achieving complete recovery after corticosteroid treatment. To the best of our knowledge, this represents the second reported case of MOGAD following the mRNA-1273 vaccine and the first presenting as isolated and unilateral optic neuritis.

## Introduction

The COVID-19 pandemic, caused by the SARS-CoV-2, emerged in late 2019. Neurological involvement has been reported as a consequence of either direct viral invasion or para-/post-infectious aberrant inflammatory responses, including autoantibody production [1,2]. Several neuro-ophthalmological complications, isolated or as part of generalized neurological syndromes, have been associated with both SARS-CoV-2 infection and vaccination [1,2].

The mRNA-1273 vaccine, developed by Moderna and the Vaccine Research Center at the National Institute of Allergy and Infectious Diseases, has demonstrated significant clinical efficacy and safety against COVID-19 [3]. Common adverse events are typically mild and include transient headache, injection site pain, muscle spasms, and myalgia [3]. However, a few cases of central nervous system (CNS) demyelinating diseases following mRNA vaccinations have been reported [4-8].

Myelin oligodendrocyte glycoprotein antibody-associated disease (MOGAD) is now recognized as an independent immune-mediated inflammatory CNS disease, characterized by the presence of anti-myelin oligodendrocyte glycoprotein (MOG) antibodies in serum or cerebrospinal fluid (CSF) [9]. Post-vaccine MOG antibody positivity, particularly following SARS-CoV-2 immunization, is rare [10]. We report the case of a 28-year-old female who developed acute unilateral optic neuritis following SARS-CoV-2 vaccination.

This article was previously presented as a poster at the 2022 European Neuro-Ophthalmology Society Congress, held from June 20 to 23, 2022, in Cambridge, UK.

## Case presentation

**Figure 1:**
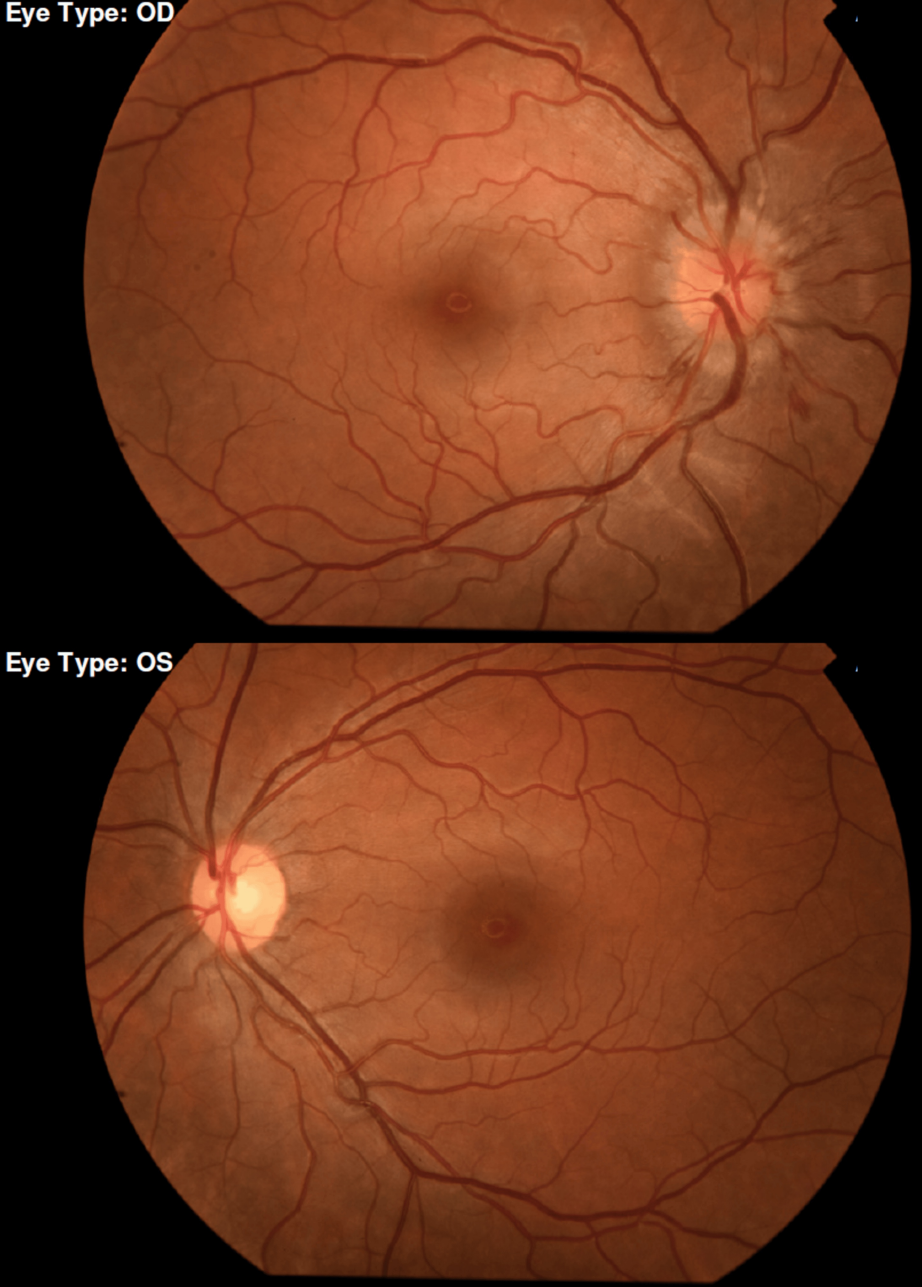
Fundus photography revealing optic disc edema in the OD, with associated flame-shaped hemorrhage and no macular involvement (upper image); OS fundus (lower image) without changes. OD, right eye; OS, left eye

A 28-year-old female presented to the emergency department with a five-day history of retro-ocular pain in her right eye (OD), exacerbated by eye movements, which began seven days after receiving the first dose of the Moderna COVID-19 vaccine (mRNA-1273). Over the preceding two days, she had experienced blurred vision in OD, with gradual spontaneous improvement. On examination, visual acuity was 20/40 in OD and 20/20 in the left eye (OS), with a right afferent pupillary defect. Ocular motility, anterior segment evaluation, and intraocular pressure were normal. Fundus examination of OD revealed 360° disc edema with flame-shaped hemorrhages, with no macular involvement (Figure 1).

**Figure 2:**
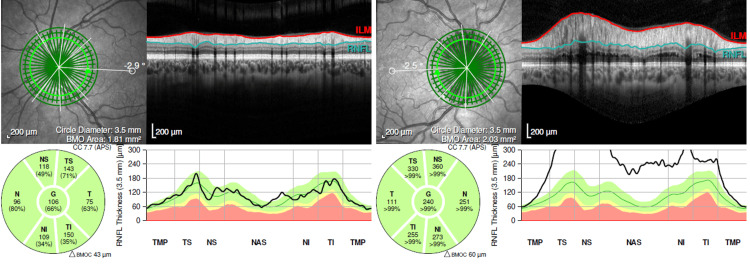
Optical coherence tomography confirming a generalized increase in peripapillary nerve fiber layer thickness in the OD (right side of the figure), with normal thickness in the OS (left side of the figure). OD, right eye; OS, left eye; G, global; N, nasal; T, temporal; NS, nasal-superior; TS, temporal-superior; NI, nasal-inferior; TI, temporal-inferior; TMP, temporal; NAS, nasal; RNFL, retinal nerve fiber layer; ILM, internal limiting membrane; BMOC, Bruch’s membrane opening complex

The posterior segment of OS was unremarkable. Optical coherence tomography showed a generalized increase in peripapillary nerve fiber layer thickness in OD (Figure 2), corresponding to an enlarged blind spot on visual field testing.

The Farnsworth-Munsell 100-Hue Test revealed blue/yellow color vision deficits and visual evoked potentials demonstrated prolonged latency with delayed conduction in the right retrochiasmal pathway.

**Figure 3:**
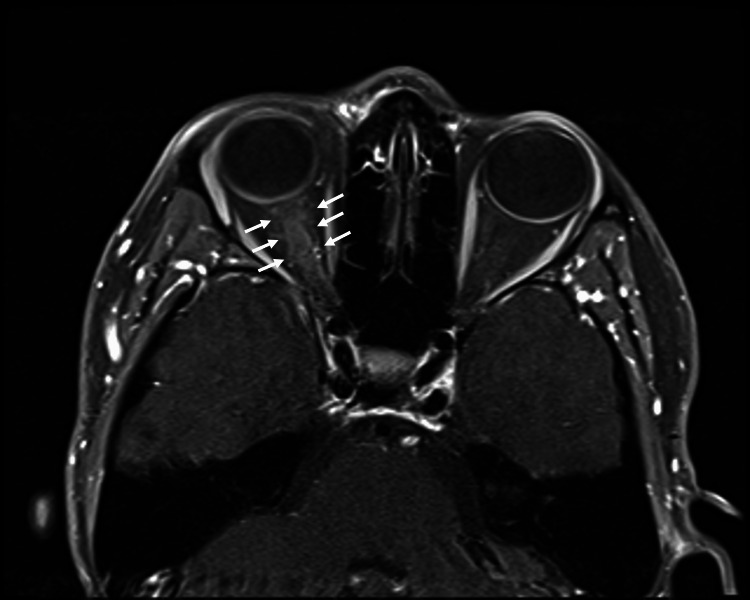
T1-weighted fat-suppressed post-gadolinium contrast orbital magnetic resonance imaging revealing right optic sheath enhancement (white arrows).

**Figure 4:**
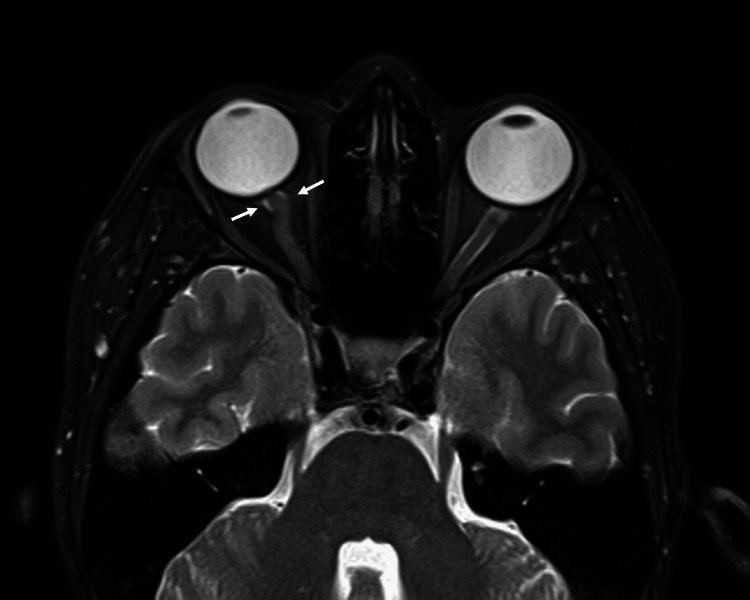
T2 hyperintensity on orbital magnetic resonance imaging showing right optic nerve sheath (white arrows).

Magnetic resonance imaging (MRI) of the brain and orbits showed T2 hyperintensity and thickening of the right optic nerve, with contrast enhancement along the nerve sheath extending from the globe to the prechiasmal segment. No brain parenchymal abnormalities were detected (Figure 3 and Figure 4).

A nasopharyngeal swab for SARS-CoV-2 by PCR was negative. Routine blood tests were unremarkable (Table 1).

**Table 1:** Summary of blood tests performed. HDL, high-density lipoprotein; LDL, low-density lipoprotein; dsDNA, double-stranded deoxyribonucleic acid; NMO, neuromyelitis optica; AQ-4, aquaporin-4; MOG, myelin oligodendrocyte glycoprotein; HIV, human immunodeficiency virus; VDRL, venereal disease research laboratory test; TPHA, treponema pallidum hemagglutination assay; HBs, hepatitis B surface; HBc, hepatitis B core; HC, hepatitis C; CMV, cytomegalovirus

Test	Result	Reference values
Hemoglobin, x10 g/L	13.6	12.0-15.0
Leukocytes, x10^9^/L	7.10	4.5-11.0
Platelets, x10^9^/L	235	150-450
Prothrombin time, seconds	12.4	9.4-12.5
Activated partial thromboplastin time, seconds	30.1	25.1-36.5
Glycose, mg/dL	98	60-100
Urea, mg/dL	15	15.0-40.0
Creatinine, mg/dL	0.76	0.57-1.11
Aspartate aminotransferase, U/L	12	5.0-34.0
Alanine transaminase, U/L	8	0.0-55.0
Gama-glutamyl transferase, U/L	9	9.0-36.0
Alkaline phosphatase, U/L	67	40-150
Total bilirubin, mg/dL	1.14	0.20-1.20
Sodium, mEq/L	140	136-145
Potassium, mEq/L	3.7	3.5-5.1
Chloride, mEq/L	107	98-107
Calcium, mg/dL	10.2	8.4-10.2
Phosphorus, mg/dL	2.6	2.3-4.7
Magnesium, mg/dL	2.02	1.60-2.60
C-reactive protein, mg/L	<0.2	<5.0
Erythrocyte sedimentation rate, mm/h	4	<16
Total proteins, g/L	72.1	60.0-83.0
Albumin, g/L	43.2	35.0-52.0
Total cholesterol, mg/dL	127	<190
HDL cholesterol, mg/dL	52	≥45
LDL cholesterol, mg/dL	67	<130
Angiotensin-converting enzyme, U/L	34	8-52
Serum folic acid, ng/mL	7.7	3.1-20.0
B12 vitamin, pg/mL	367	187-883
Anti-nuclear antibodies	Negative	-
Anti-dsDNA antibodies, UI/mL	Negative	-
Anti-neutrophil cytoplasm antibodies	Negative	-
Anti-Ro antibodies	Negative	-
Anti-La antibodies	Negative	-
Anti-Scl 70 antibodies	Negative	-
Anti-Jo antibodies	Negative	-
Anti-p antibodies	Negative	-
Anti-Sm antibodies	Negative	-
Anti-chromatin antibodies	Negative	-
Rheumatoid factor	Negative	-
Anti-NMO/AQ-4 antibodies	Negative	-
Anti-MOG antibodies	Positive	-
HIV 1+2 antibodies	Negative	-
VDRL	Negative	-
TPHA	Negative	-
HBs antigen	Negative	-
HBc antibodies	Negative	-
HC total antibodies	Negative	-
Anti-Rickesttsia conorii antibodies (IgG)	Negative	-
Anti-Rickesttsia conorii antibodies (IgM)	Negative	-
Anti-CMV antibodies (IgG)	Negative	-
Anti-CMV antibodies (IgM)	Negative	-
Anti-Borrelia burgdorferi antibodies (IgG)	Negative	-
Anti-Borrelia burgdorferi antibodies (IgM)	Negative	-
Anti-Toxoplasma gondii antibodies (IgG)	Negative	-
Anti-Toxoplasma gondii antibodies (IgM)	Negative	-

CSF analysis revealed a normal opening pressure (14 cmH2O), white blood cell count (1/µL), glucose level (58 mg/dL), and protein level (18 mg/dL). CSF viral serologies were negative, as were aquaporin-4 (AQP4) and MOG IgG. However, serum MOG IgG was positive (fixed cell-based assay method).

A diagnosis of MOG-associated unilateral optic neuritis following SARS-CoV-2 vaccination was established. The patient received systemic corticosteroid therapy, starting with 1000 mg of intravenous methylprednisolone for five days, followed by one month of oral prednisolone (1 mg/kg) with gradual tapering. Complete visual recovery was achieved. One year later, the patient’s visual acuity was 20/20 bilaterally, with mild temporal optic nerve pallor in OD and normal visual fields.

## Discussion

The clinical presentation of MOG-IgG seropositivity, papillitis, and extensive optic nerve involvement strongly supports the diagnosis of MOG antibody-associated optic neuritis. Additionally, the absence of other plausible causes and the latency between vaccination and symptom onset aligns with previously reported cases of post-vaccination optic neuritis [11]. Although bilateral involvement is more common in MOG-antibody-associated optic neuritis, unilateral cases have been reported, including those linked to SARS-CoV-2 infection and vaccination [7].

The link between immunization and demyelinating CNS manifestations is well-documented, though data specific to COVID-19 vaccines remains limited to case reports, making the incidence of such events difficult to estimate [11-13]. Among post-vaccination neuro-ophthalmological complications, optic neuritis is the most frequently reported [1]. Proposed mechanisms include cytokine production, alteration or expression of surface antigens, induction of novel antigens, molecular mimicry, or polyclonal activation of B cells [1]. In MOG-antibody-associated optic neuritis, vaccination may also disrupt the blood-brain barrier, activating T-cells and triggering autoantibody production. However, since the antibody status was unknown prior to vaccination, it remains unclear whether the vaccine initiated antibody production or merely unmasked a preexisting subclinical autoimmune condition [4].

To date, only a few cases of MOGAD following SARS-CoV-2 vaccination have been reported [4-8,10,14], with optic neuritis accounting for approximately 30% of these cases. Most were linked to the vector-based AstraZeneca/Oxford vaccine (ChAdOx1-S/ChAdOx1nCOV-19) and the mRNA-based Pfizer-BioNTech vaccine (BNT162b2). To our knowledge, this is the second reported case of MOGAD associated with the mRNA-1273 and the first presenting with isolated unilateral optic neuritis. The mRNA vaccine may activate nonspecific or specific cellular immunity and cytokine production, leading to blood-brain barrier disruption and subsequent MOG-IgG entry and pathogenesis [15]. In this case, the seven-day latency between vaccination and onset falls within the reported range of six to 45 days [4-8,10,14].

Recognizing this entity is crucial, as timely diagnosis enables prompt treatment. Systemic corticosteroids remain the standard of care and often result in favorable outcomes, underscoring the immune-mediated nature of the disease. In some reported cases [4-8,10,14], high-dose intravenous methylprednisolone alone was insufficient, requiring adjunctive plasmapheresis. Overall, most patients achieved at least partial recovery, with complete recovery in isolated optic neuritis cases, as seen in our patient [5].

The need for long-term immunosuppressive or immunomodulatory therapy depends on the degree of recovery and the occurrence of relapses. In this case, the patient achieved an excellent response to treatment with no relapses observed to date, eliminating the need for long-term therapy.

## Conclusions

This case highlights the importance of recognizing MOG antibody-associated optic neuritis as a rare but significant neuro-ophthalmological complication following SARS-CoV-2 vaccination. While such adverse events are rare, they emphasize the need for vigilance in post-vaccination settings, especially in cases presenting with new-onset visual symptoms. Early diagnosis and prompt initiation of systemic corticosteroid therapy are essential to optimize outcomes, as MOG-associated optic neuritis often responds favorably to timely treatment. Our patient’s complete recovery underscores the value of early intervention and thorough investigation to rule out alternative causes.

Although the exact mechanism linking COVID-19 vaccination to MOG antibody-associated disease remains unclear, the potential for vaccines to unmask or trigger autoimmune processes warrants further research. This case adds to the growing body of evidence on post-vaccination complications, emphasizing the importance of continued vigilance and investigation into rare adverse events, ensuring patient safety while maintaining confidence in vaccine efficacy.
